# Silicon Oxycarbide Thin Films Produced by Hydrogen-Induced CVD Process from Cyclic Dioxa-Tetrasilacyclohexane

**DOI:** 10.3390/ma18122911

**Published:** 2025-06-19

**Authors:** Agnieszka Walkiewicz-Pietrzykowska, Krzysztof Jankowski, Jan Kurjata, Rafał Dolot, Romuald Brzozowski, Joanna Zakrzewska, Paweł Uznanski

**Affiliations:** 1 Polish Academy of Sciences, Centre of Molecular and Macromolecular Studies, Sienkiewicza 112, 90-363 Lodz, Poland; jan.kurjata@cbmm.lodz.pl (J.K.); rafal.dolot@cbmm.lodz.pl (R.D.); romuald.brzozowski@cbmm.lodz.pl (R.B.); joanna.zakrzewska@cbmm.lodz.pl (J.Z.); 2 Technical Department, Jacob of Paradies University, Chopina 52, 66-400 Gorzow Wielkopolski, Poland; kjankowski@ajp.edu.pl

**Keywords:** microwave remote hydrogen plasma, chemical vapor deposition, octamethyl-1,4-dioxatetrasilacyclohexane, poly(oxybisdimethylsilylene), SiOC films, preceramic material

## Abstract

Silicon oxycarbide coatings are the subject of research due to their exceptional optical, electronic, anti-corrosion, etc., properties, which make them attractive for a number of applications. In this article, we present a study on the synthesis and characterization of thin SiOC:H silicon oxycarbide films with the given composition and properties from a new organosilicon precursor octamethyl-1,4-dioxatetrasilacyclohexane (^2^D_2_) and its macromolecular equivalent—poly(oxybisdimethylsily1ene) (POBDMS). Layers from ^2^D_2_ precursor with different SiOC:H structure, from polymeric to ceramic-like, were produced in the remote microwave hydrogen plasma by CVD method (RHP-CVD) on a heated substrate in the temperature range of 30–400 °C. SiOC:H polymer layers from POEDMS were deposited from solution by spin coating and then crosslinked in RHP via the breaking of the Si-Si silyl bonds initiated by hydrogen radicals. The properties of SiOC:H layers obtained by both methods were compared. The density of the cross-linked materials was determined by the gravimetric method, elemental composition by means of XPS, chemical structure by FTIR spectroscopy, and NMR spectroscopy (^13^C, ^29^Si). Photoluminescence analyses and ellipsometric measurements were also performed. Surface morphology was characterized by AFM. Based on the obtained results, a mechanism of initiation, growth, and cross-linking of the CVD layers under the influence of hydrogen radicals was proposed.

## 1. Introduction

Silicon carbide (SiC) is an inexpensive abrasive material that has been produced on an industrial scale by the high-temperature reaction of silica and carbon since the late 19th century. Today’s interest in this material is related to new methods of its production based on pyrolysis of silicon pre-ceramic polymers, mainly poly(carbosilanes), and new areas of application [1,2]. The use of silicon polymer precursors has the advantage of reducing the manufacturing temperature and ease of shape formation. This enables the production of materials in the form of fibers, layers, porous substances, as well as sensors and detectors [3]. Silicon carbide class materials such as SiOC, SiCN, and SiOCN [4,5] doped with oxygen and/or nitrogen atoms have been quite well recognized and significantly expand the possibilities of SiC applications [6]. One of the areas of intense scientific and application interests in organosilicon precursors is thin-film manufacturing technologies [7]. An example is coatings obtained from these compounds on non-metallic and metallic surfaces, showing, e.g., excellent mechanical and barrier properties. High-quality silicon carbide class layers, i.e., without undesirable cracks that occur during sintering of pre-ceramic polymer coatings, can be obtained by several methods, primarily by chemical (CVD) [8,9] or physical (PVD) vapor deposition of low-molecular weight silicon precursors [10].

Based on the previous studies using linear hydrosilanes [11,12], it can be expected that the use of cyclic precursors having Si, C, O, and H atoms and Si-Si groups in their structure will enable the production of a new class of thin silicon oxycarbide coatings with unique properties resulting from their high hardness, adhesion and excellent ability to coat the surface of various substrates with a complex profile.

In this paper, we presented the fabrication and characterization of thin films of the SiOC:H class with a given composition and properties from a new organosilicon precursor: octamethyl-dioxa-tetrasilacyclohexane (^2^D_2_). Cyclic methylsilaether ^2^D_2_ can also be polymerized by classical cationic polymerization [13,14]. The obtained poly(methylsilaethers), (-SiMe_2_)_2_O-)_n_, have a linear poly(dimethylsilane) structure, in which siloxane groupings Si-O-Si regularly separate identical Si-Si silylene sequences (oxysilylenes are silicon analogs of ethers and can therefore be referred to as silaethers). They are classified as pre-ceramic polymers with potentially more favorable properties than the commonly used poly(methylsilanes), (-SiMe_2_-)_n_, and poly(methylsiloxanes), (-Me_2_SiO-)_n_ [1]. Until now, thin films of the SiOC class from cyclic silaethers have not been obtained. Their specificity and uniqueness in the synthesis of ceramic materials can be appreciated using selective methods of producing thin films, such as remote hydrogen plasma (RHP) CVD. SiOC materials are most often obtained by catalytic cross-linking of siloxane polymers in the presence of a non-removable Pt catalyst, or more recently by the hydride transfer reaction catalyzed with borate B(C_6_F_5_)_3_ [14]. The search for new mechanisms of cross-linking, a key process in the ceramization of poly(siloxanes), in combination with the RHP-CVD polymerization method offers the possibility of obtaining pre-ceramic materials from silaether precursors.

**Figure 1:**
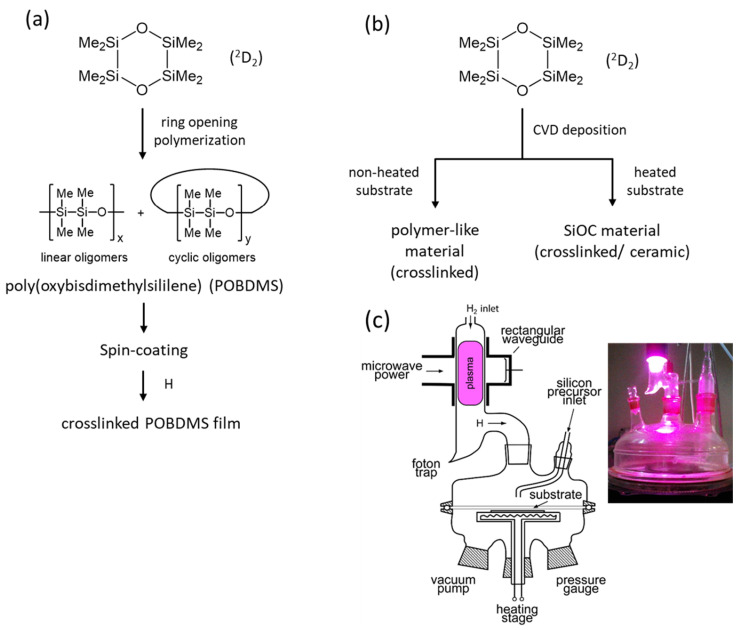
Schematic diagram of SiOC:H class coatings produced from cyclic octamethyl-1,4-dioxa-tetrasilacyclohexane (2D2) in this study: (**a**) by classical ring-opening polymerization, and (**b**) by RHP-CVD deposition. Thin films of poly(oxybisdimethylsilylene), POBDMS, were prepared by spin-coating and then cross-linked in the presence of H radicals. (**c**) Schematic diagram of the apparatus for the remote hydrogen plasma CVD process and a photograph of the actual system at work.

The presented research begins with the synthesis of cyclic silaether precursor ^2^D_2_ and permethylated polysilaether—poly(oxybisdimethylsily1ene) (POBDMS) by classical cationic polymerization. Then, the following three groups of coatings are described: (a) RHP-CVD polymer-like layers from ^2^D_2_ precursor on an unheated substrate at T_S_ = 30 °C; (b) RHP-CVD layers on a heated substrate in the temperature range up to 400 °C; and (c) cross-linked POBDMS polymer layers prepared by *spin-coating* method and then subjected to RHP treatment as a result of hydrogen attack on the Si-Si silyl sequences of the macromolecule. The reaction is selective, in which other bonds are not activated by atomic hydrogen. The structures and properties of the layers obtained in this way were compared with polymer-like SiOC: H layers obtained by the RHP-CVD methods. The concept of this research is presented in Figure 1.

This study also provides a good opportunity to show the potential of permethylated silaether polymers as compounds of PDCs (*polymer derived ceramics*), both produced by CVD polymerization and classical cationic polymerization.

## 2. Materials and Methods

Spin- and CVD-coated SiOC:H films were prepared from poly(oxybisdimethylsilylene), POBDMS, and octamethyl-1,4-dioxa-2,3,5,6-tetrasilacyclohexane, ^2^D_2_, respectively. These compounds were synthesized in our laboratory [13]. Cyclic ^2^D_2_ was obtained by hydrolytic condensation of 1,2-dichlorotetramethyldisilane. The monomer was purified by repetitive crystallization and sublimation (melting point 45 °C). ^29^Si NMR showed a singlet at 3.93 ppm, ^1^H NMR a triplet at 0.215 ppm, and ^13^C NMR a signal at 2.44 ppm. Octamethyl-1,4-dioxacyclohexasilane forms colorless crystals as a result of sublimation and crystallization on the walls of the vial. Detailed crystal data and XRD studies are presented in the Supporting Information (Figure S1). The ^2^D_2_ precursor ampoule was degassed prior to use in the CVD process.

POBDMS was prepared by the cationic ring-opening polymerization of ^2^D_2_ initiated with trifluoromethanesulfonic acid in methylene chloride at 25 °C as described in ref [15,16]. The polymerization was carried out for 8 h in a glass reactor purged with dry nitrogen, having a stopcock for withdrawing samples. An appropriate amount of CF_3_SO_3_H in CH_2_Cl_2_ was introduced by a syringe to obtain the initiator concentration 5 × 10^−3^ mol L^−1^. After 8 h, 85% conversion of monomer had been achieved. The polymerization was then quenched with a 5:1 mol/mol mixture of Et_3_N and Me_3_SiCl. Et_3_N was used in a 10-fold molar excess to the initiator. Me_3_SiCl was added to the silylate end groups. The polymer was precipitated in methanol, the solvent was evaporated, and the polymer was further dried by heating at 90 °C under vacuum (10^−2^ Torr) for 20 h. The purity of the synthesized poly(oxybisdimethylsilylene) was confirmed by the ^1^H, ^13^C, and ^29^Si NMR spectra in CDCl_3_, which showed only one sharp singlet at 0.1443 ppm, 1.983 ppm, and 0.772 ppm, respectively. The polymer had a unimodal molecular weight distribution of M_n_ = 35,000 with M_n_/M_w_ = 2.5. The basic physical properties of the POBDMS polymer have been studied previously and indicate a mesophase structure at 25 °C [17].

SiOC:H thin films from ^2^D_2_ precursor were deposited in the microwave-assisted CVD reactor as previously described in detail [15,18]. Briefly, the system consists of three sections: a glass tube, the upper part of which is coupled to microwaves (2.45 GHz) and is used to generate atomic hydrogen. The lower part is a remote section and is equipped with a Wood’s horn photon trap. The tube is connected to a CVD reactor through a center conical joint (29/32). The CVD reactor consists of two flat flange lids (HWS Labortechnik, Mainz, Germany) with nominal widths of 20 cm, having also side sockets, sealed with an O-ring. A heated stainless-steel substrate holder (13 cm in diameter) was placed in the lower lids. A source compound injector (4 mm in diameter) is located at the inlet of the hydrogen stream approximately 4 cm in front of the substrate. The distance between the plasma edge and the substrate was ~40 cm. No deposition was observed during the CVD process in the remote section, indicating that there was no back diffusion of a source compound into the hydrogen supply tube. The monomer was fed from an ampoule through a tube heated to a temperature of T = 50–60 °C. The CVD reactor was evacuated using a two-stage rotary pump (40 m^3^ h^−1^, Leybold, Model TRIVAC D40B, Cologne, Germany). The chamber pressure was measured with a resistive manometer (Pfeiffer Vacuum, Model TPR 280, Wetzlar, Germany) and monitored via the TPG 262 unit. The samples were deposited with the following parameters: hydrogen gas flow rate *F*(H_2_) = 100 sccm, monomer flow rate *F*(^2^D_2_) = 2.2 mg min ^−1^, total pressure during deposition *p* = 380 Pa (2.85 Torr), and input power of 2.45 GHz microwave plasma P = 70 W. The hydrogen flow was regulated by a mass flow controller (MKS, Herdecke, Germany) and the monomer flow by a needle valve. The coatings were produced in the substrate temperature range *T_S_* = 30–400 °C on p-type c-Si (100) wafers for the infrared, XPS, ellipsometric analysis, and nanoindentation tests and on Fisher microscope glass (20 × 20 × 0.2 mm) for the film mass determination.

Fourier transform infrared (FTIR) absorption spectra of the coatings were measured in normal transmission mode using a JASCO FTIR-6200 spectrometer (Tokyo, Japan) with a nitrogen purge.

The chemical structure of the samples was investigated by ^29^Si and ^13^C solid-state CP/MAS NMR measurements with proton decoupling using a Bruker Model Avance III WB 400 MHz NMR spectrometer (Billerica, MA, USA) at a frequency of 79.5 and 100.6 MHz for ^29^Si and ^13^C nuclei, respectively. CP cross polarization contact time was 10 ms for both ^1^H−^29^Si and ^1^H−^13^C CP/MAS NMR experiments. The sample rotation frequency was 8 kHz. Spectra were collected a few days after deposition. The films measured by CP/MAS NMR were deposited on an unheated silicon wafer substrate for about 350 min. The resulting material (approx. 150 mg) was then scraped off and analyzed.

Surface chemical characterization of the SiOC films was carried out using an AXIS Ultra photoelectron spectrometer (XPS, Kratos Analytical Ltd., Manchester, UK) equipped with a monochromatic Al–Kα X-ray source (1486.6 eV). The power of the anode was set at 150 W, and the hemispherical electron energy analyzer was operated at a pass energy of 20 eV for all high-resolution measurements. Measurements were carried out with a charge neutralizer. The main component of the C 1s line, assigned to C-C/C-H and set to 284.6 eV, was used to calibrate the spectra.

The surface morphology of the films was studied by atomic force microscopy (AFM) on c-Si substrates using a Nanoscope IIIa Digital equipped with a JV scanner operating in tapping mode (Veeco, Santa Barbara, CA, USA). The surface roughness was characterized by the root mean square R_rms_ of the laser beam position, which is equal to the statistical deviation of the vertical displacement component of the blade.

Photoluminescence (PL) spectra were recorded at room temperature by means of a Horiba Jobin Yvon (Oberursel, Germany), Fluorolog 3–22 spectrofluorimeter using an excitation wavelength of 350 nm.

The thickness and optical properties of the ceramic coatings deposited on the c-Si wafers were obtained using a variable angle spectroscopic ellipsometry (VASE) with a J. A. Woollam V-VASE ellipsometer (Lincoln, IL, USA). The ellipsometric angles psi (Ψ) and delta (Δ) were measured in the range 260–1000 nm with a step of 5 nm, at three incidence angles of 65°, 70°, and 75°, at 20 revolutions of the analyzer to average each measured point signal for all samples. To obtain the values of thickness and optical parameters *n* (λ) and k (λ), i.e., refractive index (RI) and extinction coefficient, respectively, the Cauchy–Urbach dispersion equation was used as a model for fitting to ellipsometric data. The film density was calculated based on independently determined mass and thickness values measured gravimetrically and ellipsometrically, respectively.

Thermogravimetric measurements of the changes in the SiOC:H deposit mass as a function of temperature were performed using a TGA 5500 device (TA Instruments, New Castle, DE, USA) in the range of 30–1000 °C and a heating rate of 10 °C/min in nitrogen flow. Thermal analysis of the samples was performed by differential scanning calorimetry (DSC) by heating them from −50 °C to 350 °C at a rate of 10 °C/min using a DSC 2920 calorimeter (TA Instruments, USA).

Single crystal XRD measurements were performed at −173 °C on a Rigaku Oxford Diffraction XtalAB Synergy diffractometer (Rigaku Europe SE, Neu-Isenburg, Germany) equipped with a CuKα radiation (λ = 1.5406 Å λ = 1.5418 Å) using the CrysAlisPro (Rigaku Oxford Diffraction, v.1.171.41.123a, 2022) control program. Powder X-ray diffraction (PXRD) was performed on a Malvern Panalytical Empyrian (Almelo, the Netherlands) instrument operating in a θ–2θ acquisition scan and using monochromatic radiation from a CuK_α_ source with a wavelength of λ = 1.5406 Å operating at a voltage of 45 kV and a current of 40 mA.

The summary of analytical methods used in the work is included in Table 1.

## 3. Results and Discussion

### 3.1. Properties of HRP-Treated POBDMS Coatings

**Figure 2:**
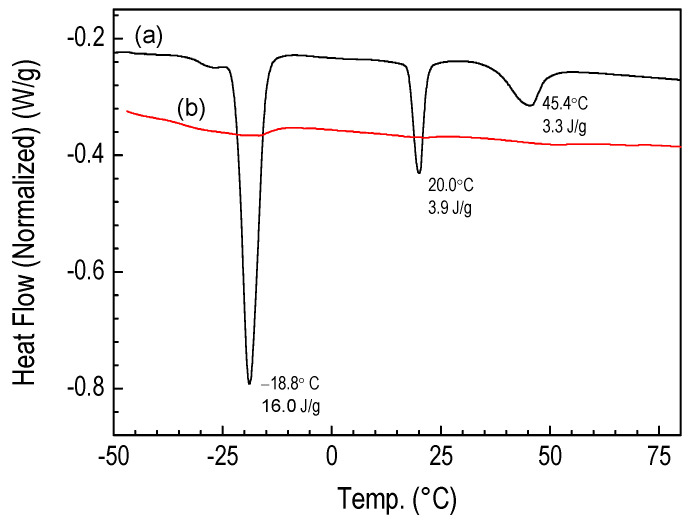
DSC thermograms of the POBDMS sample after synthesis (**a**) and HRP-POBDMS sample after hydrogen treatment at 120 °C for 15 min (**b**).

The most suitable temperature conditions for reactions with atomic hydrogen are those in which there is no crystalline phase or mesophase, i.e., in the molten state. The thermogram of the POBDMS sample is presented in Figure 2, which shows in the measured temperature range three first-order transitions: a crystal-to-crystal transition at −18.8 °C, a crystal-to-mesophase transition at 20 °C, and a broad transition to the isotropic melt at 45.7 °C (Figure 2a) [17]. Therefore, at conditions above 55 °C, the sample remains in a viscous, molten liquid state and is more susceptible to reactions with hydrogen. The polymer coating reacted with hydrogen radicals in RHP at 120 °C for 5 min, hereinafter referred to as the HRP-POBDMS sample, does not show any transformation in the temperature range from −50 °C to 80 °C. (Figure 2b). This indicates a great change in the structure of the RHP-POBDMS thin film as confirmed by XRD measurements.

**Figure 3:**
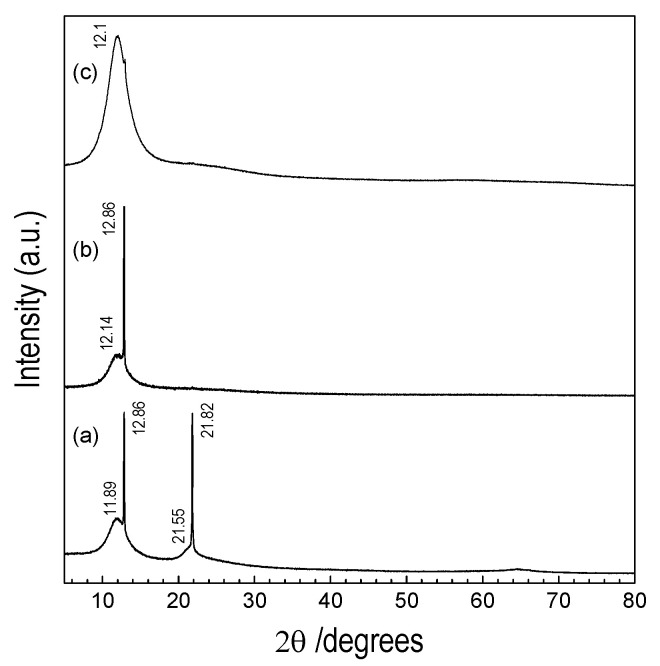
XRD patterns of POBDMS thin films: (**a**) as-synthesized sample, (**b**) heated to 120 °C for 5 min, (**c**) after treatment as-synthesized sample in microwave remote hydrogen plasma for 15 min at 120 °C.

The XRD pattern of POBDMS casted from CH_2_Cl_2_ shows at 25 °C two broad and relatively weak maxima at 2θ = 11.89° and 21.55°, and two narrow and very intense peaks at 2θ = 12.86° and 21.82° (Figure 3a). The reflections in the first group correspond to Bragg distances of about 7.5 Å and 4.1 Å and are due to the high contrast between the chain backbone of the polymer and the side groups [17]. The reflections of the second group correspond to chain axis lateral packing distances of 6.9 Å and 4.1 Å, respectively.

Heating the POBDMS sample at 120 °C for 5 min and slow cooling causes the disappearance of the peak at 21.82° together with its low-intensity counterpart at smaller angles (Figure 3b). The structure transforms to the simplest ordered state with only one very intense peak at 12.86°, and the peak corresponding to amorphous packing of chains at 12.14°. The diffraction pattern of the POBDMS layer exposed to hydrogen radicals does not show any crystalline peak, but only a broad feature at 2θ = 12.1° attributed to the amorphous content of the sample (Figure 3c). This means that the crystalline structure of the polymer is lost after HRP treatment.

### 3.2. Film Deposition Rates in RHP-CVD Process from ^2^D_2_ Precursor

**Figure 4:**
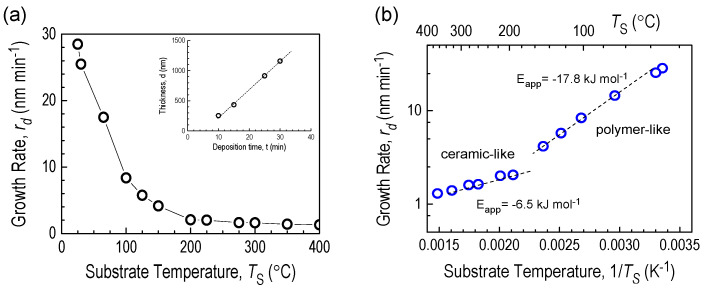
(**a**) Growth rates of the a-SiOC CVD layer deposited from the 2D2 precursor as a function of substrate temperature. (**b**) Growth rates r_d_ in Arrhenius activation coordinates. The inset in figure (**a**) is a plot of the deposition thickness vs. time for T_s_ = 30 °C.

Deposition of films from the source compound ^2^D_2_, ((Me_2_Si)_2_O)_2_, by the RHP-CVD method took place in an environment free from excited hydrogen atoms and UV photons isolated in a photon trap. This trap has the form of a horn at the end of the pipe feeding hydrogen to the reactor. The excited hydrogen atoms in the microwave cavity mainly emit light with wavelengths of 100.0, 486.2, and 655.9 nm [19,20]. Hydrogen-activated molecules in the gas phase, mainly in the form of radical species, were deposited on a temperature-programmed substrate. It was found that the deposition rate r_d_ for a given T_S_ is independent of the deposition time, and for T_S_ = 30 °C it is r_d_ = 28 nm min^−1^. The r_d_ value decreases with increasing substrate temperature, and for T_S_ = 350 °C, it is only 1.4 nm min^−1^. Changes in the rate of film formation on a substrate heated to 400 °C are shown in Figure 4a. These data are also plotted in Arrhenius coordinates (Figure 4b) to determine the thermal activation parameters of CVD deposition.

As can be seen from the graph, the measurement points of the deposition rate are arranged along two straight lines, the slopes of which change in the temperature range of 180–200 °C. The activation parameters calculated from the linear relationships of these graphs, i.e., the values of the apparent thermal activation energy (E_app_), resulting from the slope of r_d_ on the Arrhenius diagram, are −17.8 kJ mol^−1^ and −6.5 kJmol^−1^. This suggests that although the activation of the source compound occurs mainly in the gas phase, the deposition process is controlled by adsorption of film-forming products on the growth surface.

E_app_ = E_r_ + ΔH_ads_

The two temperature ranges of deposition rates may indicate the presence of two film growth mechanisms. The slowdown in layer thickness growth observed at higher temperatures is influenced by thermally activated reactions leading to the formation of volatile products and an increase in layer density. These phenomena are responsible for the observed negative activation energy E_app_ of film-forming reactions on the growth surface. Thus, the negative values of E_app_ are due to the fact that the absolute value of ΔH_ads_ is greater than the value of E_r_, where E_r_ is the activation energy of the film-forming reaction, and ΔH_ads_ is the apparent heat of adsorption of film-forming precursors on the growth surface and has a negative value. The small absolute values of E_app_ in CVD processes indicate that the adsorption and surface reactions only slightly exceed diffusion to the growth surface. The negative values of the apparent activation energy for ^2^D_2_ (Figure 4b) are caused by the higher concentration of film-forming precursors in the gas phase, determining the adsorption mechanism of the CVD process.

### 3.3. Chemical Structure of the CVD Films

#### 3.3.1. FTIR Studies

**Figure 5:**
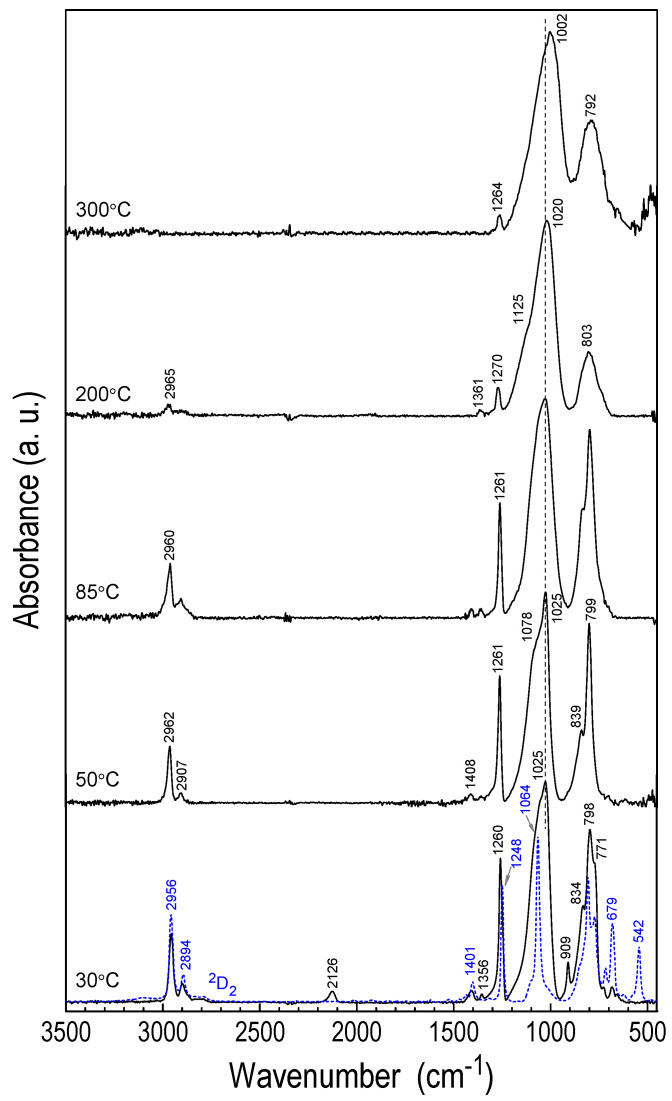
FTIR transmission spectra of RHP-CVD films deposited from ^2^D_2_ precursor at different substrate temperatures (blue line ^2^D_2_ monomer spectrum).

The film’s structure as a function of the deposition temperature was studied by FTIR absorption spectroscopy (Figure 5). IR spectroscopy of CVD thin films is one of the most useful techniques for obtaining information about their molecular composition and structure [21]. As a result of the selective reaction of the ^2^D_2_ precursor with H^•^·and its conversion to molecular species involved in the formation of a thin film, IR absorption spectra characteristic of silicon oxycarbide-like material were recorded with increasing temperature. Depending on the deposition temperature, IR spectra differ in the intensity of the bands originating from the Si-H and Si-CH_n_ bonds (1 ≤ *n* ≤ 3) and in the shape of the bands related to the C-Si-O group structure. At higher temperatures of the T_S_ substrate (>180 °C), cross-linked structures with a low content of organic groups begin dominate over polymer-like structures obtained at low temperatures up to ~150 °C (Figure 5). The assignment of the observed absorbance bands in FTIR spectra is given in Table 2. It is based on literature IR data for known organosilicon compounds [22,23,24], as well as for CVD layers studied from other precursors in previous work [18,25]. A detailed analysis of these spectra indicates that not only the deposition rate changes with temperature, but also the structure of the CVD films.

The IR spectrum of the ^2^D_2_ precursor shows vibrations characteristic of the methyl siloxane group at 2956 and 2894 cm^−1^ (stretching vibrations ν(C-H)), 1400 and 1248 cm^−1^ (bending vibrations δ(Si-CH_3_)), in the range of 900–700 cm^−1^ (multimode stretching vibrations ν(Si-C)) and at 681 and 664 cm^−1^ rocking vibrations ρ(CH_3_). For siloxane bond, the ν(Si-O-Si) stretching band is visible at 1068 cm^−1^ and 542 cm^−1^ (vibrations of the Si-Si-O- cycle structure) (Figure 5, Table 2). In the case of the layer deposited at T_S_ = 30 °C, the last IR band at 542 cm^−1^ disappears, and the Si-O-Si band is clearly expanded in the range of 1200–960 cm^−1^. It contains three spectral features located at ~1120, ~1065 and 1026 cm^−1^, respectively.

The shoulder at 1065 cm^−1^ corresponds to the Si-O-Si stretching vibrations as in disiloxanes, and its position indicates the existence of a significant number of short Si-O-Si units. On the other hand, the shoulder at 1120 cm^−1^ may indicate the presence of longer and/or branching structures. The maximum at 1026 cm^−1^ may correspond primarily to the newly formed Si-CH_2_-Si disilylmethylene unit [26], and not to the Si-O-C moiety, as it correlates with the new 1365 cm^−1^ band attributed to deformation vibrations δ(CH_2_) [27]. In addition, two new bands were observed at 2122 and 910 cm^−1^ associated with the formation of Si-H bonds. These bands are a sign of breaking the Si-Si bond in the ^2^D_2_ molecule in the presence of hydrogen radicals. The opening of the ^2^D_2_ ring is also accompanied by a shift in vibrations δ(Si-CH_3_) from 1248 cm^−1^ to 1259 cm^−1^, which is attributed to a decrease in the number of methyl substituents on the Si atom.

The absorption bands between 900 and 700 cm^−1^ are overlapping vibration modes of different groups of atomic bonds. The three superimposed maxima at 834, 798, and 774 cm^−1^ can be attributed to the -CH_3_ rocking vibrations in Si-(CH_3_)_x_ and the C-Si-O and Si-C stretching vibrations. In fact, these bands, with the exception of Si-H peaks, are observed for layers deposited up to T_S_ = 180 °C. Above this temperature, a drastic decrease in the intensity of the CH bands at ~2950 cm^−1^ and ~1260 cm^−1^ is observed, with a simultaneous restructuring of the dominant wide bands at 1002 and 792 cm^−1^, which is attributed to Si-O-Si (left side of the band at 1125 cm^−1^), Si-C-Si, and Si-O-C vibrations. As can be concluded from the lack of the -OH band at ~3500 cm^−1^, deposited layers are not very sensitive to moisture present in the air. In our previous work, we observed a similar relationship. For the films deposited from the 1,1,3,3-tetramethyldisiloxane (TMDSO) precursor, the temperature-induced transition from the SiOC polymer-like material structure with a large number of -CH_3_ units to a ceramic layer with high cross-link density and low hydrogenation degree occurred at much lower T_S_ temperature, i.e., at ~100 °C [12].

A strong decrease in the intensity of the maximum associated with the Si-CH_3_ group at 1274 cm^−1^ is observed above the substrate temperature of 180 °C and may be related to a change in the mechanism of layer growth involving free radical processes, such as Si-C, C-H and Si-Si bond cleavage. A significant part of the methyl groups must be converted into skeletal silylmethylene groups, and at higher temperatures into carbide groups. At the same time, a deep modification of the band shape is observed, mainly related to Si-O-Si/Si-C-Si bonds. A decrease in the intensity of the band shoulder at 1150 cm^−1^ and an increase in the band maximum at 1005 cm^−1^ are other manifestations of large changes in the structure of the forming layer. Moreover, as the deposition temperature increases, the shape modification is more pronounced, accompanied by an increase in the layer density [11,28], as will be discussed later in this paper.

**Figure 6:**
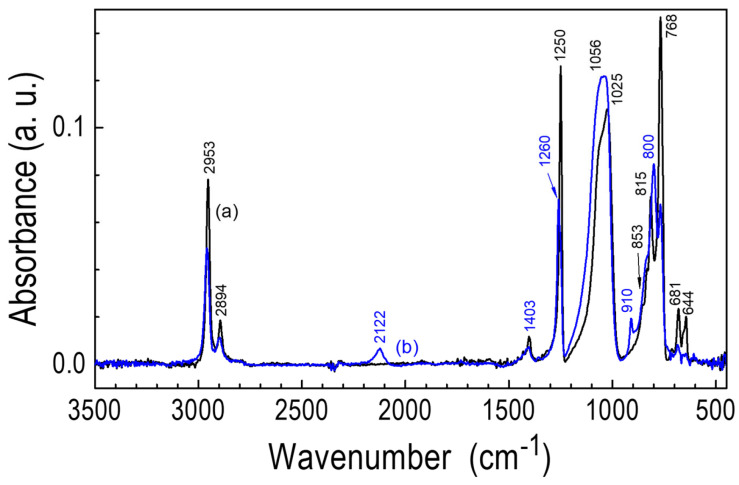
IR transmission spectra of the spin-coated film from POBDMS (a) and after RHP treatment for 20 min. at 120 °C (b).

The IR spectrum of the POBDMS polymer is similar to that of the ^2^D_2_ monomer, except for the shift in the Si-O-Si band from 1064 to 1025 cm^−1^ and its broadening, and the absence of the band at 542 cm^−1^ (Figure 6). These differences are due to the cyclic and linear structure of the compounds and their molecular weights.

Exposure of the polymer to H radicals at a temperature of 120 °C initiates a reaction with the Si-Si bonds to form Si-H bonds, absorbing at 2124 (Si-H stretching vibrations) and 910 cm^−1^ (Si-H bending). Moreover, the position of the Si-O-Si band maximum changes from 1025 cm^−1^ to 1056 cm^−1^ due to the increase in its intensity on the higher frequency side. A relative change in the intensity of maxima in the 900–750 cm^−1^ band and the disappearance of two maxima at 681 and 644 cm^−1^ in relation to the original POBDMS are also observed. As a result, the spectrum of the restructured POBDMS coating becomes similar to the spectrum of the CVD layer (it has the same spectral features except for the band at 1356 cm^−1^) deposited at T_S_ = 30 °C from ^2^D_2_.

#### 3.3.2. ^29^Si and ^13^C NMR Spectroscopy of ^2^D_2_ CVD Films and RHP-Treated POBDMS Coatings

The structural characteristics of the fabricated SiOC:H materials were supplemented by experiments of high-resolution solid-state ^29^Si and ^13^C NMR spectroscopy (Figure 5). Measurements in solution were only for a small, soluble portion of CVD products and will not be presented here. NMR studies were carried out for CVD layers deposited at 30 °C on the unheated substrate, as only this material could be collected from the substrate. Layers produced at slightly higher temperatures (75–100 °C) were quite hard and adhered strongly and could not be mechanically removed.

Solid-state CP/MAS NMR enables precise monitoring of the environment of silicon atoms, in particular the presence of oxygen and carbon atoms. For branched and partially cross-linked SiOC:H CVD deposits with a fairly large number of organic groups (CH_n_, 1 ≤ *n* ≤ 3), NMR analysis shows their complex chemical structure, significantly different from the precursors. Both for CVD films obtained on an unheated substrate and POBDMS coatings exposed to hydrogen plasma, a mixture of at least three of the five possible tetrahedral combinations of SiO_x_C_4−x_ (x = 1, 2, 3) is observed in the ^29^Si NMR spectrum. This is the result of the restructuring of the Si-O and Si-C bonds after breaking the Si-Si bonds and modifying the silicon atom environment [29].

**Figure 7:**
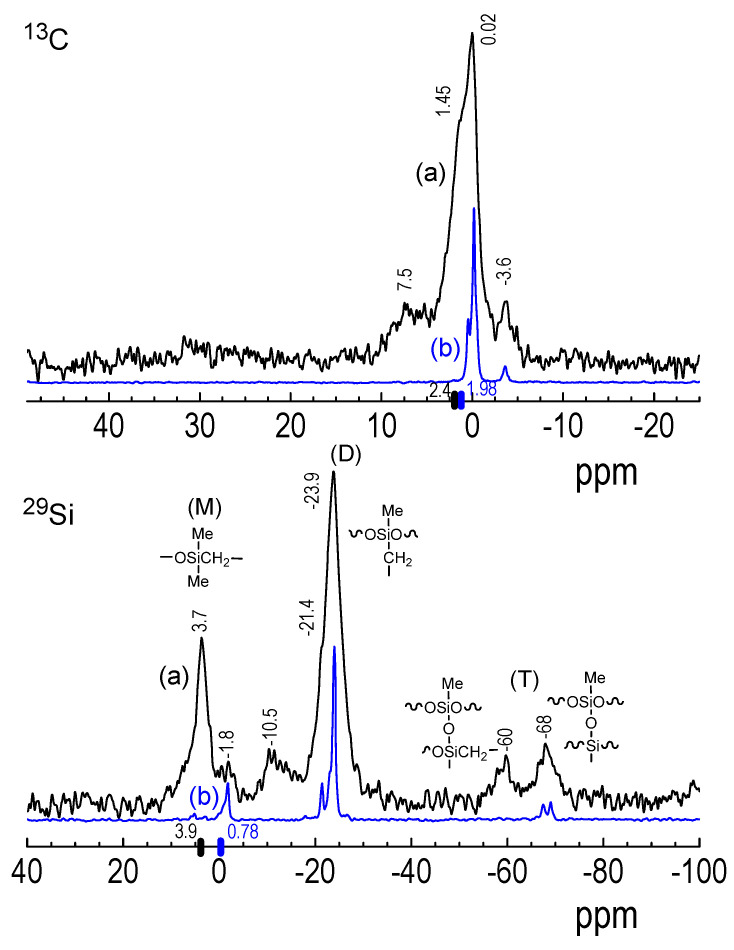
^29^Si CP/MAS NMR (**bottom**) and ^13^C CP/MAS NMR (**top**) spectra of CVD material deposited at 30 °C (a, black line) from cyclic ^2^D_2_ and from linear POBDMS after hydrogen plasma at 120 °C (b, blue line). The vertical bars on the chemical shift axes indicate the resonances for the starting ^2^D_2_ monomer and POBDMS.

The ^29^Si NMR spectrum of the cyclic ^2^D_2_ precursor in CDCl_3_ contains only one intense signal at 3.93 ppm, which is attributed to the methylated silicon atom, whereas for POBDMS this resonance is shifted towards the higher field at 0.78 ppm. Similarly, ^13^C NMR signals are in a slightly different position—at 2.44 ppm (^2^D_2_) and 1.98 ppm (POBDMS). In the carbon NMR spectrum, these signals are attributed to Si(CH_2_)_2_ units and terminal Si–(CH_3_)_3_ groups, the concentrations of which are initially high. In the CP/MAS NMR spectra of the CVD deposit, these signals are still visible, but with a significantly reduced intensity (Figure 7). At the same time, new dominant and smaller, broad signals are observed, which can be attributed to the structural M units (SiOC_3_) at 3.7 ppm, M^H^ (SiOHC_2_) at −1.8, −10.5 ppm, D (SiO_2_C_2_) at −23.9 ppm, T (SiO_3_C) at −60 and −68 ppm, where M is the silicon atom bonded to one oxygen atom, D to two, and T to three O atoms, whose position is usually observed at −10 ppm and below −100 ppm, respectively [7,30]. On the other hand, at −23.9 ppm, new structural units containing the Si-CH_2_-group appear, which are responsible for the cross-linked structure of the material already at low temperatures.

The complex ^29^Si NMR spectrum of H-treated POBDMS (at 120 °C) in Figure 7 indicates a strong restructuring of the POBDMS chain. The weak Si-Si bond is easily homolytically cleaved by H^•^, leading to the depolymerization of the linear chain, resulting in the formation of linear, cyclic, or branched oligomeric and cross-linked products. The ^29^Si spectrum of RHP-POBDMS corresponds to that of a CVD sample at 30 °C, i.e., it shows resonance signals at −1.8, −21.4, −23.0, and −24.0 ppm, but no signals at 3.7, −10.5, and −60 ppm. On the other hand, ^13^C NMR signals at 7.5, 1.48, 0.02, and −3.6 ppm have their counterparts for RHP-POBDMS at 0.45, −0.18, and −3.62 ppm (without the resonance at 7.5 ppm). For the CVD deposits, they are clearly wider than for the RHP-POBDMS coatings after reaction with hydrogen.

#### 3.3.3. XPS Studies of ^2^D_2_-CVD Films and POBDMS RHP-Treated Coatings

The aim of the XPS analyses was to obtain further detailed data on the changes in the chemical structure of ^2^D_2_-CVD films as a function of deposition temperature and the structure of the POBDMS RHP-coating modified by hydrogen plasma to compare it with the ^2^D_2_-CVD formed on an unheated substrate. Note that XPS measurements only concern the surface layer, a few nanometers thick, and not the composition throughout the entire cross-section of the film.

**Figure 8:**
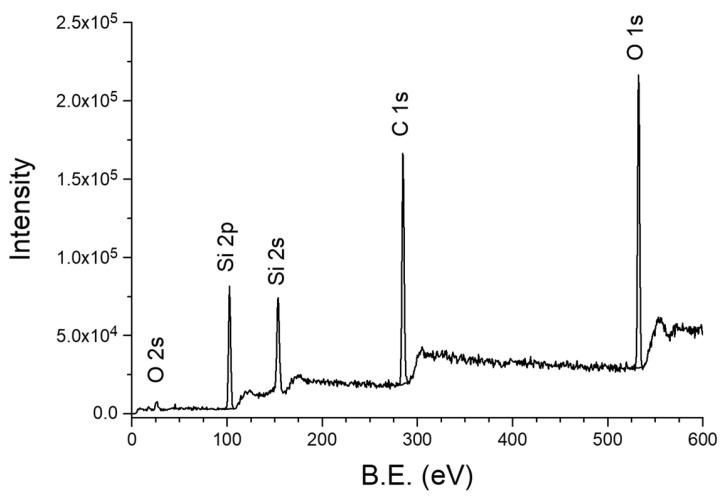
XPS survey spectrum of the RHP-CVD layer deposited from ^2^D_2_ at 120 °C. XPS survey spectrum of a ^2^D_2_ film deposited at 120 °C by the RHP-CVD method.

**Figure 9:**
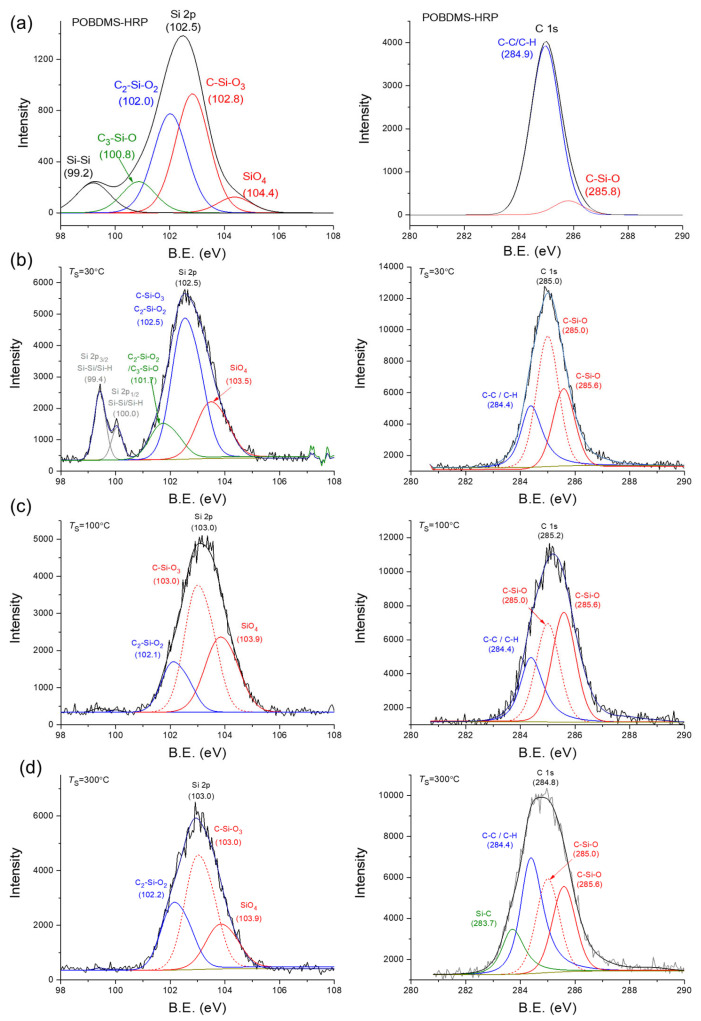
XPS Si 2p and C 1s wide-scan spectra of (**a**) POBDMS after RHP treatment at 120 °C and CVD thin films deposited from ^2^D_2_ at 30 °C (**b**), 100 °C (**c**), and 300 °C (**d**).

An example of the XPS survey spectrum (wide-scan) of the tested films (Figure 8) shows the presence three peaks at the bond energy of 101.2, 282.6, and 530.5 eV, which correspond to the energy levels of the core electrons (from the inner shells) of silicon, carbon and oxygen atoms with the Si 2p, C 1s, and O 1s configuration [31]. High-resolution XPS band analysis was performed for the Si 2p and C 1s bands, which were resolved into components associated with different chemical environments of the atoms. For example, the XPS Si 2p band can be decomposed into three components with the binding energies of 101.9, 102.6, and 103.6 eV resulting from the configuration of the Si atom with two (-C_2_-Si-O_2_-), three (-C-Si-O_3_-), and four (-Si-O_4_-) oxygen atoms, respectively. In turn, in the XPS C 1s spectra for samples deposited at higher temperatures (T_S_ = 300 °C), the number of C-C/C-H components decreases in favor of components with a different carbon neighborhood, i.e., Si-C and -C-Si-O- (Figure 9).

XPS spectra of the polymer-like layers, i.e., obtained by the RHP-CVD method from ^2^D_2_ at 30 °C and POBDMS RHP-treated coating by *spin coating*, followed by cross-linking in a remote hydrogen plasma (RHP) at 120 °C, contain a Si 2p band with electron-binding energies 99.4/100.0 eV assigned to the components Si 2p_3/2_/Si 2p_1/2_; for POBDMS-RHP, one wide band at 99.2 eV is visible. The presence of these bands comes from the Si 2p electrons involved in the Si-Si bonding, which decay at higher temperatures after H attack/insertion. The wide, intense Si 2p band at 102.5 eV for the CVD−30 °C film can be resolved into three components of 101.7, 102.6, and 103.5 eV, and for the POBDMS -RHP sample into 4 components of 100.6, 102.0, 102.8, and 104.4 eV (Figure 9). The proportion of the 102.5 eV high-energy band in the XPS spectrum is greater for the CVD sample obtained at 30 °C than for the POBDMS-RHP sample, indicating a loss of Si-Si bonds (see below). The 102.5 eV band is characteristic of dimethylsiloxane polymers and is attributed to the Si-O/C-Si-O bond (silicon oxide/silicon oxycarbide). The maximum of the Si 2p band for samples deposited at temperatures above 100 °C moves to the position at 103.0 eV. The C 1s spectrum for the POBDMS-RHP sample after deconvolution is described by the main component derived from the C-C/C-H bonds, as well as for the ^2^D_2_-CVD−30 °C sample, with the dominant component from the C-Si-O moiety. As the deposition temperature increases, the proportion of carbon moieties does not decrease, which is evident by the composition of both the Si 2p and C 1s bands. This is due to the emergence of a new Si-C carbide bond manifested as a widening of the envelope on the lower energy side at 283.7 eV of the bond.

**Figure 10:**
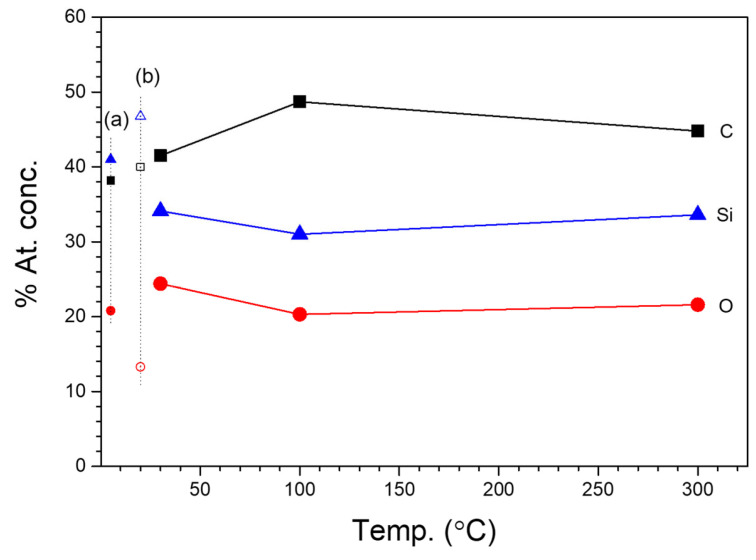
The content of individual elements measured by XPS in the surface region of the CVD layer from ^2^D_2_ as a function of the substrate temperature, T_S_. Doted vertical axis of the graph shows the content of individual C, O, and Si atoms in the HRP-POBDMS coating (a) and in the ^2^D_2_ precursor molecule (b).

Based on wide-scan XPS measurements and photoemission line intensity in the range of up to 1250 eV (sample scan in Figure 8), the elemental composition of the films deposited at different temperatures was analyzed, and their percentage content in the material from the near-surface area was determined. With increasing substrate temperature, the oxygen concentration in a deposit increases. For comparison, the content of individual C, O, and Si atoms in the ^2^D_2_ precursor molecule is marked in Figure 10 with small symbols, on the vertical axis of the graph. The composition of the a-SiOC:H layer obtained at a low substrate temperature (T_S_ = 30 °C) contains less silicon and more carbon compared to the composition of the precursor. XPS analysis shows a slight change in the dependence of atomic composition on substrate temperature. The layer-forming products contain less silicon (33.6%) and carbon (34.8%) in favor of oxygen (31.7%) compared to their content in the source precursor, which is 46.7% (Si), 40% (C), and 13.3% (O), respectively. On the other hand, the content of individual elements in the RHP-POBDMS coating is slightly different, but changes in a similar way, i.e., the silicon (41%) and carbon (38.2%)content is lower, while the oxygen (20.8%) content is higher. The increase in oxygen content in the surface layer of the RHP-POBDMS coating indicates oxidation that may occur as a result of contact of the samples with the atmosphere after RHP treatment.

#### 3.3.4. Chemical Reactions Involved in the Formation of CVD Layers from the ^2^D_2_ Precursor

Based on the structural studies characterizing the deposition products of the source compound in the reaction with atomic hydrogen and literature data describing the gas-phase reactions of disilane compounds with hydrogen radicals [25,32], a hypothetical mechanism of the activation in the studied RHP-CVD process can be proposed. Studies on the reactivity of hydrosilane compounds in the presence of atomic hydrogen have shown that the C-H, Si-C, and Si-O bonds are non-reactive, while the Si-Si bond is responsible for the observed ability of ^2^D_2_ to form layers already at the stage of initiating CVD processes. The temperature-dependent mechanism of thin film deposition from cyclic disilyl compounds, resulting from kinetic studies, involves silane precursor activation, layer growth, and cross-linking, which contribute to the formation of the a-SiOC:H material [25]. It has been shown that the Si–Si bond cleavage, Equation (1), is the first decomposition step in HRP processes. This decay can occur by frontal attack (perpendicular to the Si-Si bond) or lateral attack (along the Si-Si bond) of the hydrogen atom [33]. For the hexamethyldisilane molecule (HMDS), the frontal attack energy barrier, E_a_ = 13.4 kJ mol^−1^, is lower than the lateral attack energy barrier, E_a_ = 27.6 kJ mol^−1^. The frontal attack of the hydrogen atom on the Si-Si bond is the energetically more favored reaction. It should be noted that the energy barrier for the dissociation of the Si-H bond in the reaction with a hydrogen atom is even lower, E_a_ = 10 kJ mol^−1^.

##### Activation of Precursor Molecules

The primary reaction associated with the conversion of the ^2^D_2_ precursor to reactive products is the insertion of the hydrogen radical H^•^·to the Si-Si bond (ΔH = −24 kJ mol^−1^), initiating its dissociation [25]. This process leads to the formation of radical centers on the silicon atom. The silaether cycle, ((Me_2_Si)_2_O)_2_, has two potential reactive centers initiating ring opening and subsequent reactions that can lead to branching and/or oligomerization reactions and, at higher temperatures, to elimination of methyl groups and cross-linking of the layered deposit. An open cycle can have a radical or biradical structure with siloxane groups ((1) and (2)):

H^•^ + ((Me_2_Si)_2_O)_2_ → HMe_2_Si−O−Me_2_SiSiMe_2_−O−Si^•^Me_2_ (1)

H^•^ + HMe_2_Si−O−Me_2_SiSiMe_2_−O−Si^•^Me_2_ → Me_2_Si^•^−O−Me_2_SiSiMe_2_−O−Si^•^Me_2_ (2)

In the next step, the products (1) and (2) can recombine to ^2^D_2_, polymerize (3), or decay into shorter fragments with hydrogen attachment (4) or abstraction (5):

2(Me_2_Si^•^−O−Me_2_SiSiMe_2_−O−Si^•^Me_2_) → Me_2_Si^•^−O−(Me_2_Si)_2_O)_3_−Si^•^Me_2_ (3)

H^•^ + Me_2_Si^•^−O−Me_2_SiSiMe_2_−O−Si^•^Me_2_ → Me_2_Si^•^−O−Si^•^Me_2_ + Me_2_HSi−O−Si^•^Me_2_ (4)

H^•^ + Me_2_HSi−O−Si^•^Me_2_ → Me_2_Si^•^−O−Si^•^Me_2_ + H_2_ (5)

It can be seen that the radical products of Reactions (4) and (5) are structurally identical to the activation products of tetramethyldisiloxane (TMDSO) in the remote hydrogen plasma, and undergo similar secondary reactions as those previously proposed for this compound [11]. The biradical (5) can fragment to 1,1-dimethylsilanone and dimethylsilylene:

Me_2_Si^•^−O−Si^•^Me_2_ → Me_2_Si = O + Me_2_Si: (6)

Reaction (6) can take place both in the gas phase due to the heat released in the exothermic hydrogen recombination reaction on a biradical particle or on a heated substrate. On a heated surface, dimethylsilylene, Me_2_Si:, formed in Reaction (6), can isomerize to 1–methylsilene (7) [34]:

(7) $$Me_{2}\mathrm{Si}:\overset{\;\Delta \;}{\to }\mathrm{MeHSi}=CH_{2}$$

Dimethylsilanone, dimethylsilylene, and 1–methylsilene, the highly reactive products (6) and (7), may actively participate in the growth process of the silicon oxycarbide layer.

The compounds formed in Reactions (1) and (2) can recombine with hydrogen to form a linear tetramethylbis(dimethylsilyloxy)disilane (HMe_2_Si-O-Me_2_Si-)_2_, or with another radical product (1), to form a longer linear silaether molecule (8) or a larger cycle (9):

2(HMe_2_Si−O−Me_2_SiSiMe_2_−O−Si^•^Me_2_) → Me_2_HSi−O−(Me_2_SiSiMe_2_−O)_3_−SiHMe_2_ (8)

2(Me_2_Si^•^−O−Me_2_SiSiMe_2_−O−Si^•^Me_2_) → (Me_2_Si)_2_O)_4_) (9)

It should be noted that the products containing a hydrogen silane group, e.g., (1) and (4), can undergo sequential hydrogen elimination to form the corresponding radicals. The hydrogen radicals can react via insertion with Si-Si disilane bonds, also with the products adsorbed on the growth surface of the layer, e.g., (6), similarly as in the case of the polymeric RHP-POBDMS layer. The siloxane radicals produced in the reactions according to Equations (1) and (2) can undergo secondary reactions with the Si-Si group present in the precursor molecule ^2^D_2_ and in the products (8), (9) [35]:

~OMe_2_Si^•^ + ~OMe_2_Si−SiMe_2_O~ → ~OMe_2_SiH + ~OMe_2_Si−Si(C^•^H_2_)MeO~ (10)

The reaction product (10), the dimethylsiloxydisilane radical, can isomerize to the trimethyldisilamethylene radical (11) and then dissociate into the dimethylsiloxane radical and methylsilene (12) [11]:

~OMe_2_Si−SiMe(C^•^H_2_)O~ → ~OMe_2_Si−CH_2_−Si^•^MeO~ (11)

~OMe_2_Si−CH_2_−Si^•^MeO~ → ~OMe_2_Si^•^~ + ~OMeSi = CH_2_ (12)

Reactions (9) and (10) are endothermic and can run on a heated substrate. Two dimethylsiloxane radicals formed in reactions according to Equation (1) may also be disproportioned (13) or recombined (14), giving methylsilene or disilane moieties.

2(~OMe_2_Si^•^) → ~OMe_2_SiH + ~OMeSi = CH_2_ (13)

2(~OMe_2_Si^•^) → ~OMe_2_Si−SiMe_2_O~ (14)

Dimethylsiloxane radicals can also directly disproportionate to methylsilene radicals:

~OMe_2_Si^•^ → ~OMeSi = CH_2_ + H (15)

and further react with the silene product to trimethyldisilamethylene radicals:

~OMe_2_Si^•^ + ~OMeSi = CH_2_ → ~OMe_2_Si−CH_2_−Si^•^MeO~ (16)

~OMe_2_SiH + ~OMeSi = CH_2_ → ~OMe_2_Si−CH_2_−SiMeO~ + H (17)

Silenes, ~OMeSi = CH_2_, can produce the cyclic product 1,3-dimethyl-1,3-disiloxanecyclobutane as a result of dimerization:


(18)

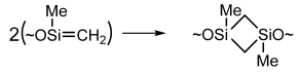



It can be concluded that the presence of linear and cyclic carbosilane fragments with the structure -Me_2_SiCH_2_SiMe_2_- is important for film growth [35].

##### Growth Reactions

The radical products of the activation Reactions (1) and (2) can recombine on the substrate’s surface to form linear or cyclic segments according to Reactions (5) and (6), which dominate at low substrate temperatures. At higher temperatures, highly reactive reaction products (3) and (4)—dimethylsilanone, dimethylsilylene, and 1–methylsilene—begin to play an important role in the propagation of layer growth through insertion into the forming layer structure.

Dimethylsilanone promotes layer growth by incorporating into Si−O−Si bonds according to Equation (19).


(19)

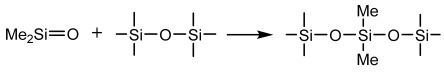



Dimethylsilylene and 1-methylsilene can readily be inserted into Si and C bonds, in methylsilyl groups, leading to branching.


(20)

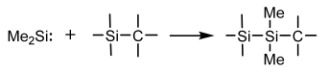




(21)

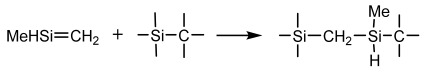



Insertion reactions of Equations (19)–(21) are exothermic and occur spontaneously. Disilane (Si-Si) bonds, on the other hand, can undergo thermal rearrangement to carbosilane bonds (-Si-CH_2_-Si-) according to Reaction (22) [36,37].


(22)

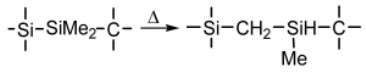



##### Cross-Linking Reactions

In growing layers on the heated substrate, methylsilyl groups and carbosilane segments may undergo thermal cross-linking according to Reactions (23) and (24).


(23)

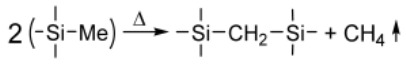




(24)

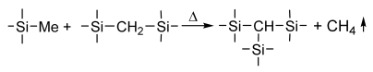



These reactions explain the decrease in the concentration of methylsilyl groups and the increase in carbosilane and carbide S-C bonds observed in IR spectra with increasing substrate temperature. Cross-linking reactions are also important for increasing the layer density. In the cross-linking process, a layer of three-dimensional structure of silicon oxycarbide is created. The reactions presented above are well confirmed by XPS results based on the concentration of the atoms forming the layer. The dependence of the silicon and carbon content is well described by the reactions that “control” the cross-linking stage. The products of the above reactions are silicon oxide or silicon oxycarbide structures. It is worth emphasizing that cross-linking occurs for a substrate temperature of T_S_ > 200 °C, because then we observe a rapid decrease in the hydrogen content caused by the elimination of methyl groups -CH_3_ in the form of methane. Carbon atoms with radicals generated during activation can remain in the cross-linked layer (Reaction (2)). Hence, the percentage content of this element decreases much more slowly than in the case of hydrogen. The observed increase in the oxygen and silicon content for substrate temperatures T_S_ > 200 °C is well described by cross-linking reactions, in which it can be seen that this layer consists mainly of oxygen and silicon atoms, devoid of organic groups.

#### 3.3.5. Thermogravimetric Studies of Polymer CVD and RHP-POBDMS Films

**Figure 11:**
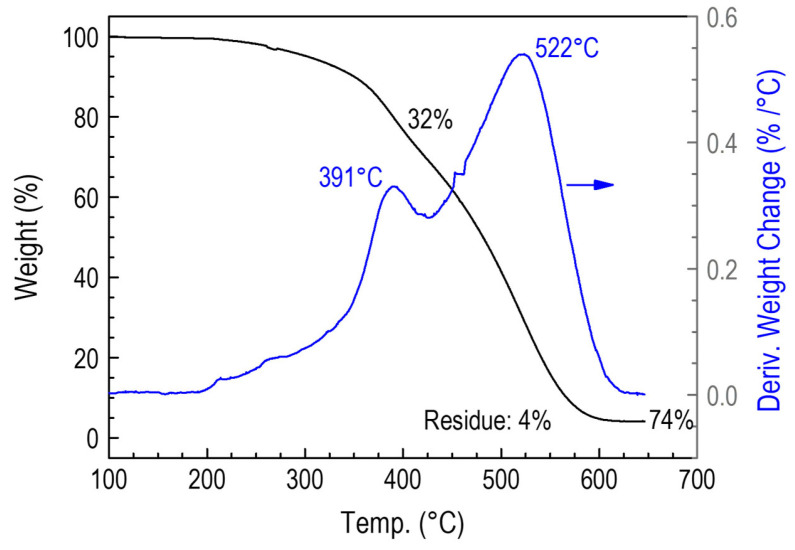
Plot of the mass change in a ^2^D_2_-CVD film formed on an unheated substrate (30 °C) during thermolysis.

**Figure 12:**
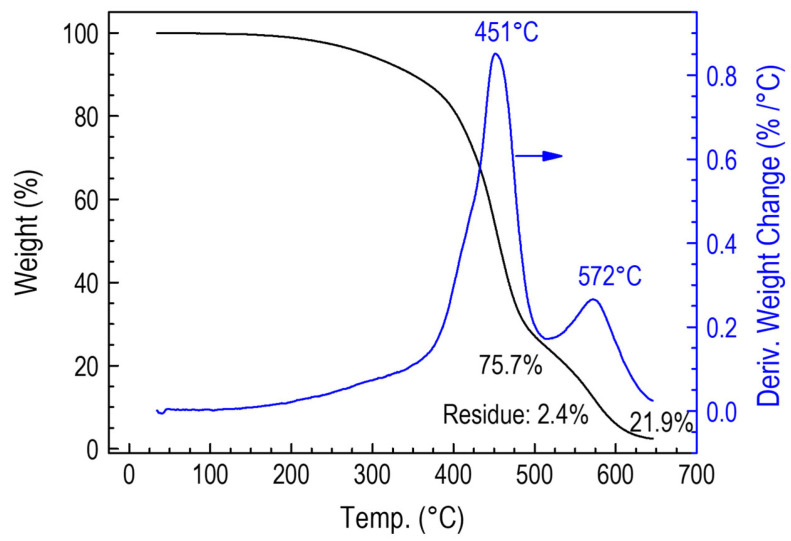
Plot of the mass change in a RHP-POBDMS coating during thermolysis.

Comparative studies were carried out on polymer layers obtained by CVD from a ^2^D_2_ precursor and a RHP-POBDMS polymer coating exposed to hydrogen plasma at 120 °C to demonstrate further differences between the two layers. Thermolysis of the ^2^D_2_-CVD deposit on an unheated substrate leads to a gradual loss of mass. The changes start at 300 °C and have two stages occurring at 391 °C and 522 °C, in which 32% and 74% of the sample weight is lost. Thermolysis is completed at 650 °C to produce solid ceramic products, silicon oxycarbide a–SiCO:H in the amount of 4 % of the initial mass (Figure 11). The sample taken at 75 °C leaves 14% residue. The thermolysis process of the cross-linked polysilaether RHP-POBDMS sample is similar. Two temperature ranges can also be distinguished: 451 and 572 °C, where the mass loss is 75.7% and 21.9%, respectively, with a residue of 2.4% at 625 °C. Thermolysis of pure POBDMS leaves 5.4% of the weight of the starting sample at 475 °C (Figure 12).

Pyrolysis in the nitrogen stream is a continuous process that leads to changes in the structure of the material and its composition. In the case of the CVD film, only changes related to high-temperature organic–ceramic conversion are observed. For the RHP-POBDMS sample, a thermogravimetric run is observed, typical for silicon oxycarbon compounds, which undergo transformations from a regular to an irregular structure at temperatures of ~200 °C. The main product of pyrolysis at 650 °C can be silica, as well as a certain amount of silicon oxycarbide with traces of amorphous carbon [38]. These products result from the homolytic cleavage of the ≡Si-CH_3_ bond (≡Si^•^∙ + ∙^•^CH_3_) and, in the presence of free radicals, the cleavage of C-H bonds, which leads to the formation of Si-CH_2_-Si, Si-CH(Si-)-Si groups, and the release of methane CH_4_.

#### 3.3.6. Analysis of Surface Morphology by AFM

**Figure 13:**
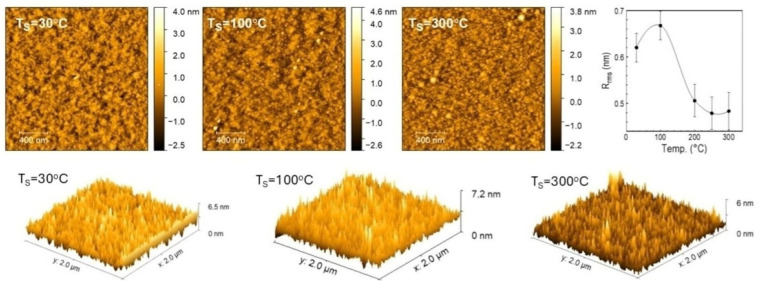
AFM microscope images of CVD layers made of ^2^D_2_ for T_S_ substrate temperatures of 30 °C, 100 °C, and 300 °C, and a graph of their surface roughness as a function of T_S_.

The morphology of the layer surface is important due to the use of CVD layers as thin coatings with a thickness of several to several dozen nanometers. Measurements with the AFM microscope show that the layers are quite smooth, homogeneous, and free from structural defects. They exhibit a granular structure, which is well characterized by the R_rms_ (root mean square) parameter (its lower value indicates greater smoothness of the layer). Sample images for three substrate temperatures, T_S_ = 30 °C, 100 °C, and 350 °C, are shown in Figure 13. 

The photos presented above show the surface irregularities of the layers. From the examination of the surface topology profile for all substrate temperatures, a diagram of the relationship between the substrate roughness R_rms_ as a function of T_S_ was obtained (Figure 13). The diagram shows that with the increase in substrate temperature, the surface roughness initially increases to 0.7 nm at 100 °C, and then decreases and approaches the value of R_rms_ = 0.45 nm for T_s_~250 °C (roughness of the c–Si/native SiO_2_ substrate is ~0.25 nm). The decrease in roughness is accompanied by the formation of finer and more numerous globular forms. At higher temperatures, larger grains appear on the surface, which are responsible for increasing the R_rms_ value. The irregular, oscillatory course of smoothness may be related to the different mobility of film-forming precursors on the growth surface as a function of the substrate temperature T_S_, which is influenced by chemical reactions causing strong cross-linking.

#### 3.3.7. CVD Film Properties—Density and Refractive Index

**Figure 14:**
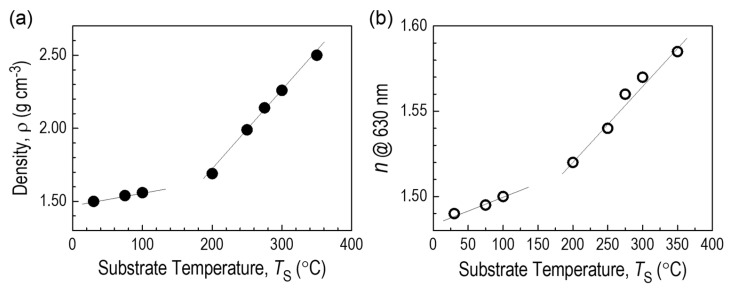
Density ρ (**a**) and refractive index *n* at λ = 630 nm (**b**) of CVD films deposited from ^2^D_2_ as a function of substrate temperature, T_S_.

The density of the layers was determined by measuring the mass of the layer deposited on a microscope slide of known surface area and thickness. The latter value was determined using ellipsometric measurements. The determined densities for different substrate temperatures T_S_ are shown in Figure 14a.

As the deposition temperature of the T_S_ layers increases, the density value increases, which indicates an increased degree of structure packing due to the elimination of organic groups, occurring at higher substrate temperatures. The increase in density caused by the cross-linking process (among others, through the formation of Si–C bonds) leads to the transformation from a polymer-like material at low temperatures to a ceramic-like material formed at higher deposition temperatures. The low density for T_S_ < 180 °C of 1.5–1.6 g·cm^−3^ may result from the low degree of cross-linking of the layer, indicating its polymer nature. A significant increase in the CVD film’s density in the substrate temperature above T_S =_ 200 °C is attributed, as already mentioned, to the layer cross-linking process. It is worth noting the abrupt change in layer thickness as a function of temperature, which occurs for T_S_ = 180–200 °C.

**Figure 15:**
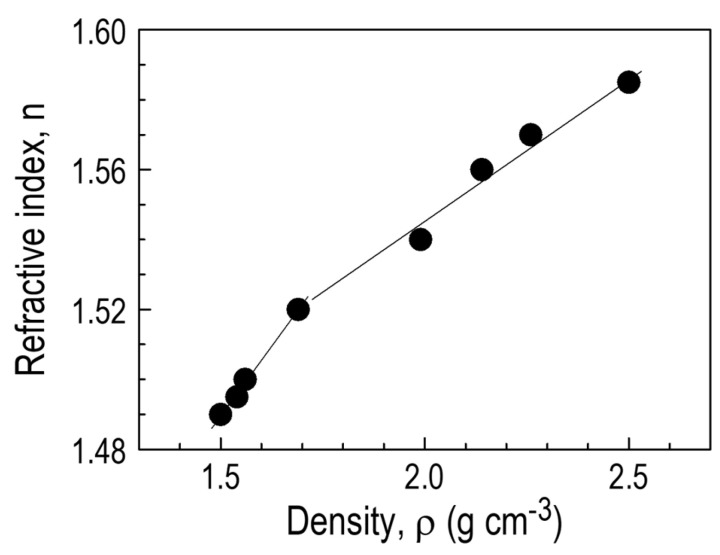
Correlation between density and refractive index of ^2^D_2_-CVD films obtained at temperatures 30–350 °C.

An important parameter in terms of the application of CVD thin films is the refractive index, which was determined in ellipsometric measurements. The obtained values of the refractive index *n* depending on the substrate temperature are shown in Figure 14b. The diagram shows that with the increase in substrate temperature T_S_, the refractive index *n* clearly increases from *n* = 1.49 at T_S_ = 30 °C to the maximum value of *n* = 1.585 for substrate temperature T_S_ = 300–400 °C. The nature of the changes is analogous to the previously discussed temperature changes in layer density with a characteristic “transition temperature” in the T_S_ range of 180–200 °C. A similar relationship is observed for density as a function of the refractive index, which allows us to conclude that *n* is closely related to layer cross-linking processes (Figure 15).

#### 3.3.8. Photoluminescence

**Figure 16:**
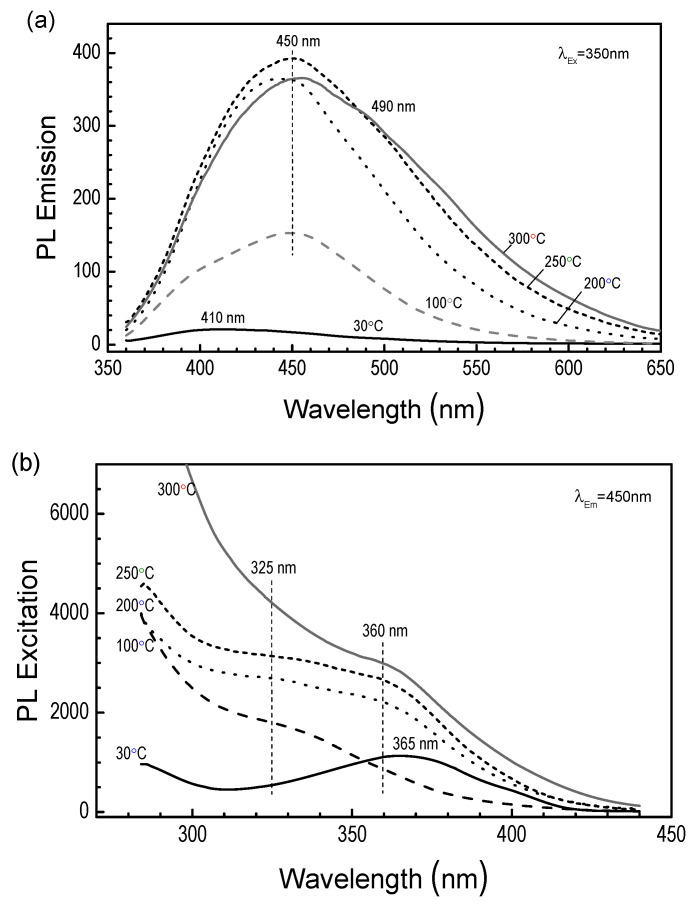
Photoluminescence (λ_Ex_ = 350 nm) spectra (**a**) and photoluminescence excitation (λ_Em_ = 450 nm) (**b**) of layers made of ^2^D_2_ for different temperatures of the T_S_ substrate.

Photoluminescence (PL) studies of the CVD layers show that they are characterized by broad emission spectra ranging from 380 to 650 nm. The results for different substrate temperatures T_S_ are shown in Figure 16a. All emission spectra are characterized by a single asymmetric shape, with a maximum in the wavelength range λ = 410–460 nm, depending on the deposition temperature. The bandwidth results from emissions that originate from various delocalized states present in the silicon-carbon a–SiOC:H layers [11]. This is indicated by photoluminescence excitation spectra (Figure 16b) illustrating the evolution of the absorption states of the layers as a function of temperature. The ^2^D_2_-CVD layer for 30 °C is characterized by a well-separated band with a maximum at 365 nm. An increase in the T_S_ deposition temperature gradually develops a new band at 325 nm and shifts the original band towards higher energies to 360 nm. There is a change in the electronic structure, which is responsible for the change in photoluminescence emission depending on the excitation energy.

## 4. Conclusions

The kinetics of layer deposition as a function of temperature show that for deposition temperatures T_S_ = 30–350 °C, the RHP-CVD process with the use of the ^2^D_2_ precursor proceeds according to two mechanisms with different activation parameters. On the basis of the results of FTIR and ^13^C and ^29^Si CP/MAS NMR studies, it was found that the layers produced in the low temperature range contain structural fragments derived from the parent precursor -(CH_3_)_2_SiO-. At high temperatures, low hydrogenation is observed, measured by the presence of CH_3_ groups. This means that the consequence of T_S_ growth is the elimination of organic groups and cross-linking through the formation of a Si-O-Si/Si-CH_x_-Si (x < 2) backbone network. The increase in deposition temperature is also accompanied by a significant increase in density (from 1.5 to 2.5 gcm^−3^) and an increase in the refractive index (from 1.49 to 1.58). These values are correlated. AFM studies of the surface showed that the a-SiOC:H layers are homogeneous with low roughness. However, their value depends variably on the temperature T_S_, first rising to 0.67 nm at 100 °C, and then falling to the lowest value of 0.3 nm at 250 °C. The layers exhibit photoluminescence, like most SiOC:H plasma materials

An interesting result seems to be the comparison of the cross-linking of a thin layer of polymer-like CVD deposit from the precursor ^2^D_2_ with a layer of silaether polymer subjected to the action of remote hydrogen plasma, POBDMS-RH. FTIR spectra indicate a mechanism of POBDMS activation in the presence of H. In the reaction with hydrogen, the -Si-Si-chain bonds break down, leading to oligomerization after hydrogen attachment, or they can recombine to form a cross-linked polymer. The presence of cross-linking is visible in the course of thermolysis of the hydrogen plasma layer. The CVD layer is expected to be more cross-linked, as evidenced by a 4.1% thermolysis residue by weight at 700 °C compared to 2.4% by weight of the POBDMS-RH residue.

## Figures and Tables

**Table 1:** Summary of characterization techniques.

Methods	Measurement
29Si and 13C CP/MAS NMR	Chemical structure
Fourier transform infrared (FTIR)	Substituent groups
X-ray Photoelectron Spectroscopy (XPS)	Atomic concentration
X-ray diffraction (XRD)	Crystallographic structure
Spectroscopic ellipsometry	Thickness and refractive index
Atomic force microscopy (AFM)	Particle size and roughness
Photoluminescence (PL)	Photoluminescence excitation and emission
Thermogravimetric analysis (TGA)	Thermal stability, decomposition, and compositional changes

**Table 2:** Assignment of IR absorption bands for a-SiOC:H layers from ^2^D_2_ for different substrate temperatures T_S_ and for the spin-coated POBDMS polymer film exposed to H^•^ radicals at 120 °C.

Band Assignment *	Monomer	CVD LayerT_S_ = 30 °C	CVD LayerT_S_ = 350 °C	POBDMS	POBDMS After RHP
ν_as_(C-H) in CH_3_	2956	2957	-	2953	2958
ν_s_(C-H) in CH_3_	2894, 2795	2901, 2796	-	2894, 2793	2897
ν(Si-H)	-	2126	-		2122
δ_as_(CH_3_) in Si-CH_3_	1401	1407	-	1403	1404
δ(CH_2_) in Si-CH_2_-Si	-	1355	-		
δ_s_(CH3) in Si-CH_3_	1248	1259	1264	1250	1260
ν_as_(SiOSi)	1064, 1026 sh Cyclic siloxanes have only 1 band	-	-	1056 sh, 1025 D type dimethylsiloxane linear chains	-
ν_as_(SiOSi), chain or small cycle, ν(Si-C-Si) in (Si-CH_2_-Si)	-	1056 sh, 1027	1133sh, ν_as_(Si-O-Si) cavity 1002 ν_as_(SiOC)	-	1056, 1033
δ(Si-H)	-	909	-	-	910
ρ(CH_3_) in Si(CH_3_)_2_	847sh	832	812sh	853, 832	853, 832
ρ(CH_3_) in the Si-Si group, ν(SiC)	806	798	792, 733sh ν(SiC) carbide	815	800
ρ(CH_3_) in Si-CH_3_	770	771	-	768	768
ν(SiC) in ((CH_3_)_2_Si)_2_ group	714, 679	728, 683	-	681	686
611 Si-Si in Crystal	-	-	-	644	-
νs(SiSiO) Si-Si-O in the ring	542	-	-	-	-

* vibration designations: ν—stretching; δ—bending; ρ—rocking; as—asymmetrical; s—symmetrical; sh—the shoulder of the band.

## Data Availability

The original contributions presented in this study are included in the article. The final structure was validated by CheckCif program (http://checkcif.iucr.org accessed on 30 May 2025) and deposited at the Cambridge Crystallographic Data Centre (CCDC) under access number 2455518. Further inquiries can be directed to the corresponding authors.

## Supplementary Materials

The following supporting information can be downloaded at: https://www.mdpi.com/article/10.3390/ma18122911/s1, Table S1. Experimental details; Table S2. Fractional atomic coordinates (×10^4^) and equivalent isotropic displacement parameters (Å^2^ × 10^3^). *U_eq_* is defined as 1/3 of the trace of the orthogonalised *U_ij_*′; Table S3. Anisotropic displacement parameters (Å^2^ × 10^4^). The anisotropic displacement factor exponent takes the form: −2*π*^2^[*h*^2^*a*^*2^ × *U*_11_ + … + 2*hka*^*^ × *b*^*^ × *U*_12_]; Table S4. Bond lengths (Å); Table S5. Bond angles (°); Table S6. Torsion angles (°); Figure S1. ^2^D_2_ structure with representation of the atoms with thermal ellipsoids at the 50% probability level. The molecule has a center of symmetry and selected bond lengths (Å) and angles (°) are as follows: Si1-Si2 2.3637(5) Å, Si1-O1 1.6431(12) Å, Si2-O1 1.6420(11) Å, Si1-C11 1.8610(17) Å, Si1-C12 1.8654(17) Å, O1-Si1-Si2 108.08(4), O1-Si2-Si1 107.46 (4), Si2-O1-Si1 144.25 (8), and C11-Si1-C12 107.95 (7); Figure S2. XRD powder diffraction pattern of the ^2^D_2_ molecule. References [39,40] have been cited in the Supplementary Materials.

## Abbreviations

The following abbreviations are used in this manuscript:

CVD	Chemical Vapor Deposition
RHP	Remote Hydrogen Plasma
AFM	Atomic Force Microscopy
PL	Photoluminescence
XPS	X-ray Photoelectron Spectroscopy
^2^D_2_	Octamethyl-1,4-dioxatetrasilacyclohexane
POBDMS	Poly(oxybisdimethylsily1ene)
RHP-POBDMS	Remote Hydrogen Plasma-treated POBDMS coatings
TGA	Thermogravimetric analysis
XRD	X-ray diffraction
