# Supplementary material for: Silicon Oxycarbide Thin Films Produced by Hydrogen-Induced CVD Process from Cyclic Dioxa-Tetrasilacyclohexane
Source: Materials (Basel). 2025 Jun 19;18(12):2911. doi: 10.3390/ma18122911 (PMC12195353; doi:10.3390/ma18122911)
Supplement: Supplementary file 1 [file materials-18-02911-s001.zip › materials-3697709-supplementary.pdf]

Supplementary Information

Silicon Oxycarbide Thin Films Produced by Hydrogen-Induced CVD Process from Cyclic Dioxa-Tetrasilacyclohexane

Agnieszka Walkiewicz-Pietrzykowska [1,*], Krzysztof Jankowski [2], Jan Kurjata [1], Rafał Dolot [1], Romuald Brzozowski [1], Joanna Zakrzewska [1] and Paweł Uznanski [1,*]

[1] Polish Academy of Sciences, Centre of Molecular and Macromolecular Studies, Sienkiewicza 112, 90-363 Lodz, Poland

[2] Technical Department, Jacob of Paradies University, Chopina 52, 66-400 Gorzow Wielkopolski, Poland

* Correspondence: agnieszka.walkiewicz-pietrzykowska@cbmm.lodz.pl (A.W.-P.); pawel.uznanski@cbmm.lodz.pl (P.U.)

1. Experimental Section

X-ray diffraction study of octamethyl-1,4-dioxa-2,3,5,6-cyclohexasilane crystals. X-ray-quality of $^2D_2$ crystals in the form of colourless, transparent prisms were formed in about 1 month at room temperature without the presence of a solvent as a result of sublimation and crystallization on the walls of the vial. A suitable crystal with dimension of 0.31× 0.31× 0.16 mm was selected and glued to the sample support using a silicone grease. *The measurements were made at 100 K on a Rigaku Oxford Diffraction XtalAB Synergy diffractometer with a CuKα radiation (λ=1.5402 Å) using the CrysAlisPro control program.* The unit cell was refined from 23984 reflections, representing 87% of the observed reflections. The final completeness of data reduction, scaling and absorption corrections is 97.5 % at Θ=75.889. The crystal structure was solved with the SHELXT program[1] and assigned to the space group $P2_1/n$. All non-hydrogen atoms were anisotropically refined into geometrically idealized positions. The final structure was validated by CheckCif program (http://checkcif.iucr.org) and deposited at the Cambridge Crystallographic Data Centre (CCDC) under access number 2455518. The data-collection, processing and refinement statistics are given in Tables S1-S6. The molecular structure of tetrasilacyclohexane has been studied previously[2], however, for the present structure the independent part was assigned differently. There is also no disorder in the area of the oxygen atoms, which was visible in the previous given structure (Figure S1).

Table S1. Experimental details.

Compound	octamethyl-1,4-dioxacyclohexasilane
**Crystal data**	
CCDC	2455518
Chemical formula	$C_8H_{24}O_2Si_4$
Formula weight	264.63
Crystal system	Monoclinic
Space group	$P2_1/n$
Temperature (K)	100.01(10)
a [Å]	7.60422(7)
b [Å]	6.40513(5)
c [Å]	16.17771(11)
β [°]	94.5127(7)
V [Å^3]	785.509(11)
Z	2
d_{calc} [g/cm^3]	1.119
Crystal dimensions [mm]	0.31×0.31×0.16

Radiation type	Cu*K*α
μ [mm^{-1}]	3.367
**Data collection**	
Reflections measured	27601
Range/indices (*h*, *k*, *l*)	-9, 8; -8, 7; -20, 20
θ (max, min) [°]	75.9, 5.5
Total no. of unique data	1596
No. of observed data, I > 2σ(I)	1586
R_{int}	0.070
**Refinement**	
R [$F^2 > 2\sigma(F^2)$]	0.034
$wR(F^2)$	0.092
S	1.13
No. of reflections	1596
No. of parameters	368
No. of restraints	0
H-atom treatment	H atoms treated by constrained refinement
Δρ (min, max), e/Å^3	-0.39, 0.42

Table S2. Fractional atomic coordinates (×10^4) and equivalent isotropic displacement parameters (Å^2 ×10^3). U_{eq} is defined as 1/3 of the trace of the orthogonalised U_{ij}.

Atom	*x*	*y*	*z*	U_{iso}*/U_{eq}
Si1	0.57886 (5)	0.35817 (6)	0.40253 (2)	0.01401 (18)
Si2	0.71261 (5)	0.54014 (6)	0.57550 (2)	0.01412 (18)
O1	0.70633 (15)	0.4199 (3)	0.48568 (8)	0.0354 (4)
C11	0.6626 (2)	0.4927 (3)	0.31151 (11)	0.0264 (4)
H11B	0.587502	0.457224	0.261450	0.040*
H11A	0.783814	0.447641	0.304894	0.040*
H11C	0.660434	0.644043	0.320243	0.040*
C12	0.5986 (2)	0.0711 (3)	0.38623 (10)	0.0201 (3)
H12B	0.534992	0.032318	0.333424	0.030*
H12C	0.547852	−0.003425	0.431619	0.030*
H12A	0.723318	0.033535	0.385087	0.030*
C21	0.8665 (2)	0.7661 (3)	0.57433 (11)	0.0249 (4)
H21C	0.882216	0.827338	0.629928	0.037*
H21A	0.817352	0.871268	0.535012	0.037*
H21B	0.980767	0.718587	0.557421	0.037*
C22	0.8026 (3)	0.3534 (3)	0.65671 (12)	0.0304 (4)
H22A	0.811332	0.422913	0.710847	0.046*
H22C	0.919885	0.306583	0.643610	0.046*
H22B	0.723751	0.232679	0.658328	0.046*

Table S3. Anisotropic displacement parameters (Å^2 ×10^4). The anisotropic displacement factor exponent takes the form: $-2\pi^2[h^2a^{*2} \times U_{11} + ... + 2hka^* \times b^* \times U_{12}]$.

Atom	U_{11}	U_{22}	U_{33}	U_{12}	U_{13}	U_{23}
Si1	0.0109 (3)	0.0223 (3)	0.0089 (2)	0.00086 (14)	0.00067 (15)	−0.00746 (14)
Si2	0.0101 (3)	0.0217 (3)	0.0099 (2)	0.00252 (14)	−0.00323 (15)	−0.00661 (14)
O1	0.0126 (6)	0.0684 (9)	0.0241 (6)	0.0099 (6)	−0.0055 (5)	−0.0318 (6)
C11	0.0316 (10)	0.0203 (8)	0.0293 (9)	−0.0042 (7)	0.0146 (7)	−0.0036 (7)
C12	0.0210 (8)	0.0240 (8)	0.0155 (7)	0.0042 (6)	0.0037 (6)	0.0012 (6)
C21	0.0157 (8)	0.0293 (9)	0.0296 (8)	0.0002 (6)	0.0006 (6)	0.0034 (7)
C22	0.0259 (9)	0.0251 (9)	0.0382 (10)	0.0003 (7)	−0.0098 (8)	0.0045 (7)

Table S4. Bond lengths (Å).

Atoms	*Length (Å)*	*Atoms*	*Length (Å)*
Si1—Si2^{i}	2.3637 (5)	C12—H12B	0.9800
Si1—O1	1.6431 (12)	C12—H12C	0.9800
Si1—C11	1.8610 (17)	C12—H12A	0.9800
Si1—C12	1.8654 (17)	C21—H21C	0.9800
Si2—O1	1.6420 (11)	C21—H21A	0.9800
Si2—C21	1.8620 (18)	C21—H21B	0.9800
Si2—C22	1.8663 (18)	C22—H22A	0.9800
C11—H11B	0.9800	C22—H22C	0.9800
C11—H11A	0.9800	C22—H22B	0.9800
C11—H11C	0.9800		

Symmetry code: (i) −*x*+1, −*y*+1, −*z*+1.

Table S5. Bond angles (°).

Atoms	*Angle (°)*	*Atoms*	*Angle (°)*
O1—Si1—Si2^{i}	108.08 (4)	Si1—C12—H12B	109.5
O1—Si1—C11	108.66 (8)	Si1—C12—H12C	109.5
O1—Si1—C12	107.70 (8)	Si1—C12—H12A	109.5
C11—Si1—Si2^{i}	111.99 (6)	H12B—C12—H12C	109.5
C11—Si1—C12	107.95 (7)	H12B—C12—H12A	109.5
C12—Si1—Si2^{i}	112.32 (6)	H12C—C12—H12A	109.5
O1—Si2—Si1^{i}	107.46 (4)	Si2—C21—H21C	109.5
O1—Si2—C21	109.27 (8)	Si2—C21—H21A	109.5
O1—Si2—C22	107.82 (8)	Si2—C21—H21B	109.5
C21—Si2—Si1^{i}	112.62 (6)	H21C—C21—H21A	109.5
C21—Si2—C22	108.03 (8)	H21C—C21—H21B	109.5
C22—Si2—Si1^{i}	111.52 (7)	H21A—C21—H21B	109.5
Si2—O1—Si1	144.25 (8)	Si2—C22—H22A	109.5
Si1—C11—H11B	109.5	Si2—C22—H22C	109.5
Si1—C11—H11A	109.5	Si2—C22—H22B	109.5
Si1—C11—H11C	109.5	H22A—C22—H22C	109.5
H11B—C11—H11A	109.5	H22A—C22—H22B	109.5
H11B—C11—H11C	109.5	H22C—C22—H22B	109.5
H11A—C11—H11C	109.5		

Symmetry code: (i) −*x*+1, −*y*+1, −*z*+1.

Table S6. Torsion angles (°).

Atoms	*Angle (°)*	*Atoms*	*Angle (°)*
Si1ⁱ—Si2—O1—Si1	6.5 (2)	C12—Si1—O1—Si2	−128.12 (19)
Si2ⁱ—Si1—O1—Si2	−6.6 (2)	C21—Si2—O1—Si1	−115.96 (19)
C11—Si1—O1—Si2	115.18 (19)	C22—Si2—O1—Si1	126.86 (19)

Symmetry code: (i) −*x*+1, −*y*+1, −*z*+1.

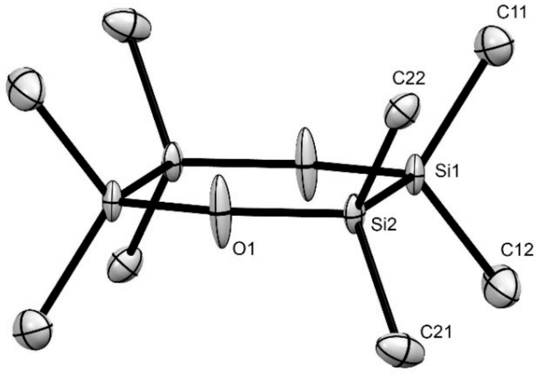

Figure S1. $^2D_2$ structure with representation of the atoms with thermal ellipsoids at the 50% probability level. The molecule has a centre of symmetry and selected bond lengths (Å) and angles (°) are as follows: Si1-Si2 2.3637(5) Å, Si1-O1 1.6431(12) Å, Si2-O1 1.6420(11) Å, Si1-C11 1.8610(17) Å, Si1-C12 1.8654(17) Å, O1-Si1-Si2 108.08(4), O1-Si2-Si1 107.46 (4), Si2-O1-Si1 144.25 (8), C11-Si1-C12 107.95 (7).

When analysing the $^2D_2$ powder diffraction pattern among many scattering peaks, there is a family consisting of 4 narrow reflections of high intensity and gradually decreasing intensity. The 2theta positions of these peaks are 10.92, 21.92, 33.10, and 44.54° (Figure S2.). The spatial separation of the reflecting layers calculated from the Bragg formula is 8.11 nm, which is half of the c-axis dimension of the cell, and perpendicular to which the molecules form the most densely packed planes.

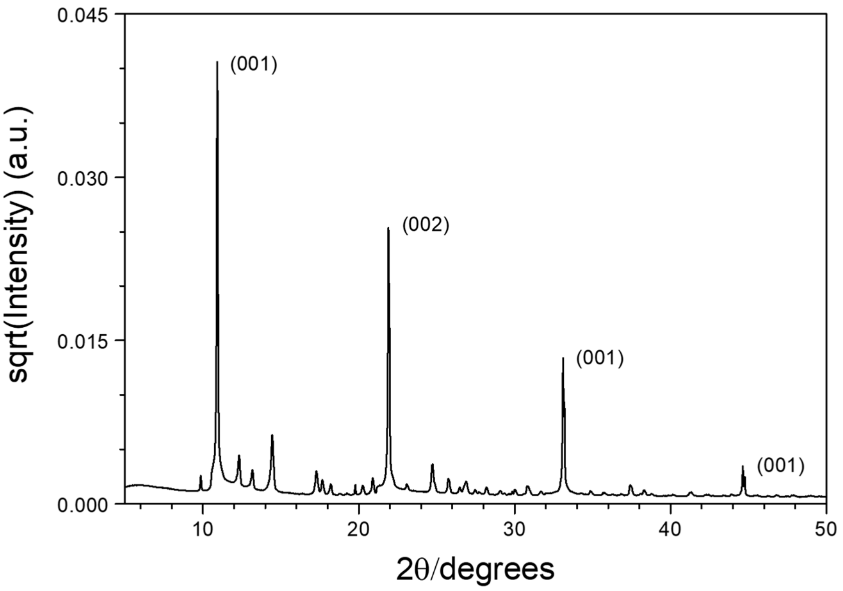

Figure S2. XRD powder diffraction pattern of the $^2D_2$ molecule.

References

1. Sheldrick, G. SHELXT—Integrated space-group and crystal-structure determination. *Acta Crystallogr. Sect. A* **2015**, *71*, 3–8.
2. Korlyukov, A.A.; Chernyavskaya, N.A.; Antipin, M.Y.; Lysenko, K.A.; Chernyavskii, A.I. Molecular and Crystal Structure of Octamethyl-1,4-dioxacyclohexasilane. *Chem. Heterocycl. Compd.* **2005**, *41*, 536–541.
